# Oroxylin A promotes retinal ganglion cell survival in a rat optic nerve crush model

**DOI:** 10.1371/journal.pone.0178584

**Published:** 2017-06-22

**Authors:** Shu-Fang Lin, Jia-Ying Chien, Kishan Kapupara, Chi-Ying F. Huang, Shun-Ping Huang

**Affiliations:** 1 Institute of Clinical Medicine, National Yang-Ming University, Taipei, Taiwan; 2 Department of Molecular Biology and Human Genetics, Tzu Chi University, Hualien, Taiwan; 3 Institute of systems neuroscience, National Tsing Hua University, Hsinchu, Taiwan; 4 Institute of Biopharmaceutical Sciences, National Yang-Ming University, Taipei, Taiwan; National Eye Centre, UNITED STATES

## Abstract

**Purpose:**

To investigate the effect of oroxylin A on the survival of retinal ganglion cells (RGC) and the activation of microglial cells in a rat optic nerve (ON) crush model.

**Methods:**

Oroxylin A (15mg/Kg in 0.2ml phosphate-buffered saline) or phosphate-buffered saline (PBS control) was immediately administered after ON crush once by subcutaneous injection. Rats were euthanized at 2 weeks after the crush injury. The density of RGC was counted by retrograde labeling with FluoroGold and immunostaining of retina flat mounts for Brn3a. Electrophysiological visual function was assessed by flash visual evoked potentials (FVEP). TUNEL assay, immunoblotting analysis of glial fibrillary acidic protein (GFAP), inducible nitric oxide synthase (iNOS) and cyclooxygenase-2 (COX-2) in the retinas, and immunohistochemistry of GFAP in the retinas and ED1 in the ON were evaluated.

**Results:**

Two weeks after the insult, the oroxylin A-treated group had significantly higher FG labeled cells and Brn3a+ cells suggesting preserved RGC density in the central and mid-peripheral retinas compared with those of the PBS-treated group. FVEP measurements showed a significantly better preserved latency of the P1 wave in the ON-crushed, oroxylin A-treated rats than the ON-crushed, PBS treated rats. TUNEL assays showed fewer TUNEL positive cells in the ON-crushed, oroxylin A-treated rats. The number of ED1 positive cells was reduced at the lesion site of the optic nerve in the ON-crushed, oroxylin A-treated group. Increased GFAP expression in the retina was reduced greatly in ON-crushed, oroxylin A-treated group. Furthermore, administration of oroxylin A significantly attenuated ON crush insult-induced iNOS and COX-2 expression in the retinas.

**Conclusions:**

These results demonstrated that oroxylin A hasss neuroprotective effects on RGC survival with preserved visual function and a decrease in microglial infiltration in the ONs after ON crush injury.

## Introduction

Traumatic optic neuropathy (TON) is a devastating cause of function impairment of vision and blindness [1]. Optic nerve injury triggers a process of degeneration in the damaged axons, possibly mediated by glial dysfunction, following the apoptotic cell signaling, retrograde axonal degeneration and Wallerian degeneration and leading to RGC apoptosis [2–4]. Numerous studies have established experimental animal models for traumatic optic neuropathy, such as optic nerve transection, stretch and crush injury models [5–8]. The optic nerve crush model can imitate the optic nerve damage and is commonly used to study neurodegenerative process in the optic nerve and retina and to screen the potential neuroprotective reagents for acute optic neuropathies. Optic nerve crush constitute a primary axonal injury which directly disrupts the axolemma, resulting in sodium and calcium influx and the activation of protease progressing to neuronal death [9–12]. Crush injury induced macrophage and microglia accumulation at the site of the insult. Reactive Muller cell and astrocyte upregulating GFAP and become hypertrophic in response to injury [13, 14]. Activated glia has been showed to release multiple inflammatory mediators, such as TNF-α, IL-6, IL-1β, MCP-1, iNOS, and COX-2 [15–19]. Nitric oxide synthase inhibitors and anti-inflammatory cytokines have been used to rescue RGC from apoptosis through inhibiting microglial activation after axon injury [20–22]. These observations have implicated neuroinflammation played a critical role in the death of RGCs after crush injury.

Oroxylin A (5,7-dihydroxy-6-methoxyflavone) is a plant-originated flavonoid isolated from medical herb *Scutellariae baicalensis Georgi*. Previous study had demonstrated that oroxylin A suppressed LPS-induced iNOS and cyclooxygenase-2 expression through inhibiting the activation of NFκB-p65 in RAW264.7 macrophages [23]. It was also reported that oroxylin A and its analogues exhibited strong inhibitory activities against LPS-induced NO production in microglia [24]. Furthermore, results from *in vivo* studies indicated that oroxylin A stimulated adult neurogenesis in the hippocampal dentate gyrus region [25], prevented cerebral hypoperfusion-induced neuronal damage [26], and ameliorated amyloid (Aβ)-induced memory impairment [27]. Oroxylin A, therefore, has been shown to exert anti-inflammatory and neuroprotective effects. In this study, the ability of oroxylin A to prevent RGC loss and preserve RGC function was examined following ON crush. We further investigated the potential molecular targets involved in oroxylin A-mediated protection and explore its role in microglial activation in ON crush model.

## Materials and methods

### Animals and ethics statement

Fifty four adult male Wistar rats weighing 150–180 grams (7–8 weeks old) were used in this study (**S1 Table**). Rats were obtained from the breeding colony at BioLASCO Co., Taiwan. The animals were maintained for at least 1 week in this environment prior to being subjected to the surgical procedures. They were maintained in cages in an environmentally controlled room that was held at a temperature of 23 ± 1°C, a humidity of 55 ± 5% and had a 12-hour light–dark cycle (light period: 7 AM to 7 PM). Rats had free access to food and water. Animal care and experimental procedures were conducted in accordance with the Association for Research in Vision and Ophthalmology (ARVO) statement for the use of animals in ophthalmic and vision research. The Institutional Animal Care and Use Committee (IACUC) at Tzu Chi University approved all animal experiments (No. 104096). A total of 54 rats were used and all rats survived until the completion of the scheduled protocols.

### Anesthesia and euthanasia

All manipulations were performed under general anesthesia induced by an intramuscular injection of a mixture of ketamine (100 mg/kg body weight (BW)) and xylazine (10 mg/kg BW; Sigma, St Louis, Mo., USA), and animals were kept warm during and after operation. In addition, topical 0.5% Alcaine eye drops (Alcon, Puurs, Belgium) were used. Topical Tobradex eye ointment (Alcon, Puurs, Belgium) was applied immediately after the surgical procedure. Animal health was monitored daily by the animal care staff and veterinary personnel. Rats were euthanized by exposure to CO_2_ at rate of 20% chamber air displacement per minute (5L/min) in a cage with wood-shaving bedding. All efforts were made to minimize suffering.

### Optic nerve crush and injury experiment

An ON crush injury was induced as described in our previous report [28]. Briefly, rats were general anesthetized and eye were further anesthetized with topical 0.5% Alcaine eye drops. The conjunctiva was cut with Vannas scissors to expose the optic nerve. Care was taken to avoid damaging the small vessels around the ON. Injury was introduced by crushing the ON 2mm posterior to the globe with a vascular clip (60 g micro-vascular clip, World Precision Instruments, FL, USA) for 30 seconds. After the surgery, Tobradex eye ointment (Alcon, Puurs, Belgium) was administered. The rats were kept under supervision and on electric heating pads at 37°C for recovery. A sham operation was performed by exposing the ON in the same way but not crushed.

### Flash visual-evoked potentials (FVEPs)

For the functional evaluation of the ON, FVEPs were recorded 2 weeks after ON crush in 18 experimental rats. We masked the groups in assessing FVEP. An isolated silver plate electrode was placed extradurally through a 2-mm diameter craniotomy over the visual cortex using the stereotactic coordinates (bregma -8 mm, lateral 3 mm) and a modified method described by Ohlsson et al.[29] We used a visual electrodiagnostic system (UTAS-E3000, LKC Technologies, Gaithersburg, MD, USA) to measure FVEPs [28, 30]. After 10 minutes of light adaptation, we performed photopic FVEP, based on the report showing no significant differences of latency between photopic and scotopic VEP in Wistar rats [31]. The settings were background illumination off, a flash intensity of Ganzfeld 0 db, single flash with flash rate on 1.9 Hz, the test average at 80 sweeps, the threshold for rejecting artifacts at 50 mV and a sample rate of 2000 Hz. We compared the latency of the first positive wave (P1) of the FVEP among groups (n = 6 in each group).

#### Retrograde labeling of RGCs with Fluorogold (FG) and densities of RGCs

The detailed procedures have been described in our previous reports [28, 32]. Briefly, we performed retrograde labeling of RGCs with FG one week before sacrificing to avoid overcounting RGCs by mixing labeled RGCs with dye-engulfed macrophages and microglia [33]. The rats were anesthetized using a ketamine (100 mg/kg) and xylazine (10 mg/kg) mixture, and then placed in a stereotactic apparatus (Stoelting,Wood Dale, IL, USA). An amount of 1.5 ml of 5% of FG (Fluorochrome,Denver, CO, USA) was injected into the superior colliculus on each side. One week after labeling, the eyeballs were harvested after euthanasia of the animals. The eyeballs were placed in 10% formalin and the whole retina was then carefully dissected, flattened. The retina was examined with a 400 epi-fluorescence microscope (Axioskop; Carl Zeiss Meditec. Inc., Jena, Germany) equipped with afilter set (excitation filter: 350–400 nm; barrier filter:515 nm), as well as a digital camera (Axiocam MRm) and software (Axiovision 4.0). The retinas were examined for RGCs at a distance of 1 or 3 mm (Fihe retinas were examined for RGCs at a distance of 1 or 3 mm from the center to provide the central and mid-peripheral RGC densities respectively. We counted eight randomly chosen areas (38250 μm^2^; 225x17 μm) in the central (about 40% of the central area) and mid-peripheral (about 30% of the mid-periphery) regions of each retina (n = 6 per group). The averages of these areas were taken as the mean density of RGCs per retina. RGC survival percentage was defined as the number of RGCs in each treatment group divided by the number of RGCs in the sham-operated retina, multiplied by 100.

### Brn3a-labeled flat-mounted retinas

Animals were euthanized and eyes were enucleated and fixed in 10% formalin for 1 hour. Retina flat mounts were subjected to immunofluorescent staining for Brn-3a as previously described [34]. In brief, flat mounts were washed with PBS and blocked with blocking buffer (PBS with 0.3% Triton-X and 5% Fetal Bovine Serum) for 1 hour. Flat mounts were then incubated with primary monoclonal antibody Brn-3a (1:200; Santa Cruz, CA, USA) for 24 hours, washed with PBS and treated with secondary antibody Alexa flour 488 (1:400; Life Technologies, OR, USA) and incubated overnight. 8 to 10 microphotographs were captured for every retina flat mount covering central and mid peripheral region.

### Optic nerve and retinal sample preparation

#### ON preparation

Segments of the ON (5–7 mm long) between the optic chiasm and the eyeball were harvested upon sacrifice at two weeks after the experiments. The nerves were immediately frozen at -70°C for future immunohistochemical studies.

#### Retinal preparation

After sacrifice, the corneas, lenses and vitreous bodies were removed. The remaining eyecups containing scleras and retinas were fixed in 4% paraformaldehyde for 2 h at room temperature. Each retinal cup was cut adjacent to the disc into two pieces. The tissues were then dehydrated in 30% sucrose overnight and kept at -20°C until further processing could be performed for sectioning.

### Terminal-deoxynucleotidyltransferase mediated nick end labeling (TUNEL) assay

To ensure the use of equivalent fields for comparison, all retinal frozen sections were prepared with retinas at 1–2 mm distance from the ONH. TUNEL reactions (DeadEnd^TM^ fluorometric TUNEL System, Promega Corporation, Madison, WI, USA) were performed to detect apoptotic cell. The TUNEL positive cells in the RGC layer of each sample were counted in ten high powered fields (HPF, x400 magnification), and three sections per eye were averaged [30].

### Immunohistochemistry (IHC) in the ONs and retina

IHC of ED1 (CD68, a marker of macrophage/microglia) in the ONs and IHC of GFAP in the retina using monoclonal antibody (ED1, 1:50; AbD Serotec, Oxford, UK) or polyclonal antibody (GFAP, 1:200; Cambridge, MA, USA) was performed. Briefly, the frozen longitudinal sections of ONs and retina were fixed with acetone at -20°C for 30min and blocked with 5% fetal bovine serum (FBS) containing 1% bovine serum albumin (BSA) for 15 min. The anti-ED1 antibody (1:50 AbD Serotec, Oxford, UK) was applied and incubated at 4°C overnight. The secondary antibody conjugated with fluorescein isothiocyanate (FITC, 1:100, Jackson ImmunoResearch Laboratories, West Grove, PA, USA) was applied at room temperature for 1h. Counterstaining was performed using DAPI (1:1000, Sigma, St. Louis, MO, USA). For comparison, ED1 positive cells were counted in six HPF at the lesion site of ON (n = 6 in each group).

### Immunoblot analysis

Total retinal protein extracts from rat retina were prepared using modified radioimmunoprecipitation (RIPA) buffer. The protein concentrations were determined using the BCA (bicinchoninic acid) protein assay kit (Pierce). Each retina was served as an individual sample (n = 3 in each group). Protein samples containing 50μg of protein were separated on 12% sodium dodecyl sulphate-polyacrylamide gels and transferred to polyvinylidene difluoride (PVDF) membranes (PerkinElmer, Waltham, MA, USA). After 30 min bloking with TBST buffer (0.02M Tris-base, pH 7.6, 0.8% NaCl, 0.1% Tween 20) supplemented with 5% dry skim milk, the membranes were incubated in anti-GFAP (1:1000; Abcam, Cambridge MA, USA), anti- iNOS (1:100; Cell signaling Inc. Beverly, MA, USA) and anti-COX-2 (1:100; Santa Cruz, CA, USA) primary antibodies at 4°C overnight. After washing, the blots were incubated in the appropriate anti-horseradish peroxidase-conjugated secondary antibody (1:10000; Bio-Rad) at room temperature for 1 h. The proteins on the membranes were detected using an enhanced chemiluminescence (ECL) system (Amersham Biosciences). The blots were also probed with an antibody for glyceraldehyde-3-phosphate dehydrogenase (GAPDH, 1:3000; Sigma-Aldrich) as an internal loading control. Densitometric analysis was conducted using ImageJ software. Each experiment was repeated three times with independent retinal samples from different animals. For comparison, the ratio of GFAP, iNOS or COX-2 signaling/GAPDH signaling on sham-operated retina was regarded as 1.0 fold.

### Statistical analysis

All measurements were performed in a masked fashion. Data are presented as the means ± standard deviation (S.D). Statistical analysis was performed with commercial software (IBM SPSS Statistics 19, International Business Machine Corp., Armonk, NY). The Kruskal-Wallis test and Mann-Whitney U test were used for comparisons between each group. In all cases, a value of p<0.05 was considered statistically significant.

## Results

### Oroxylin A administration enhanced RGC survival following ON crush

**Fig 1:**
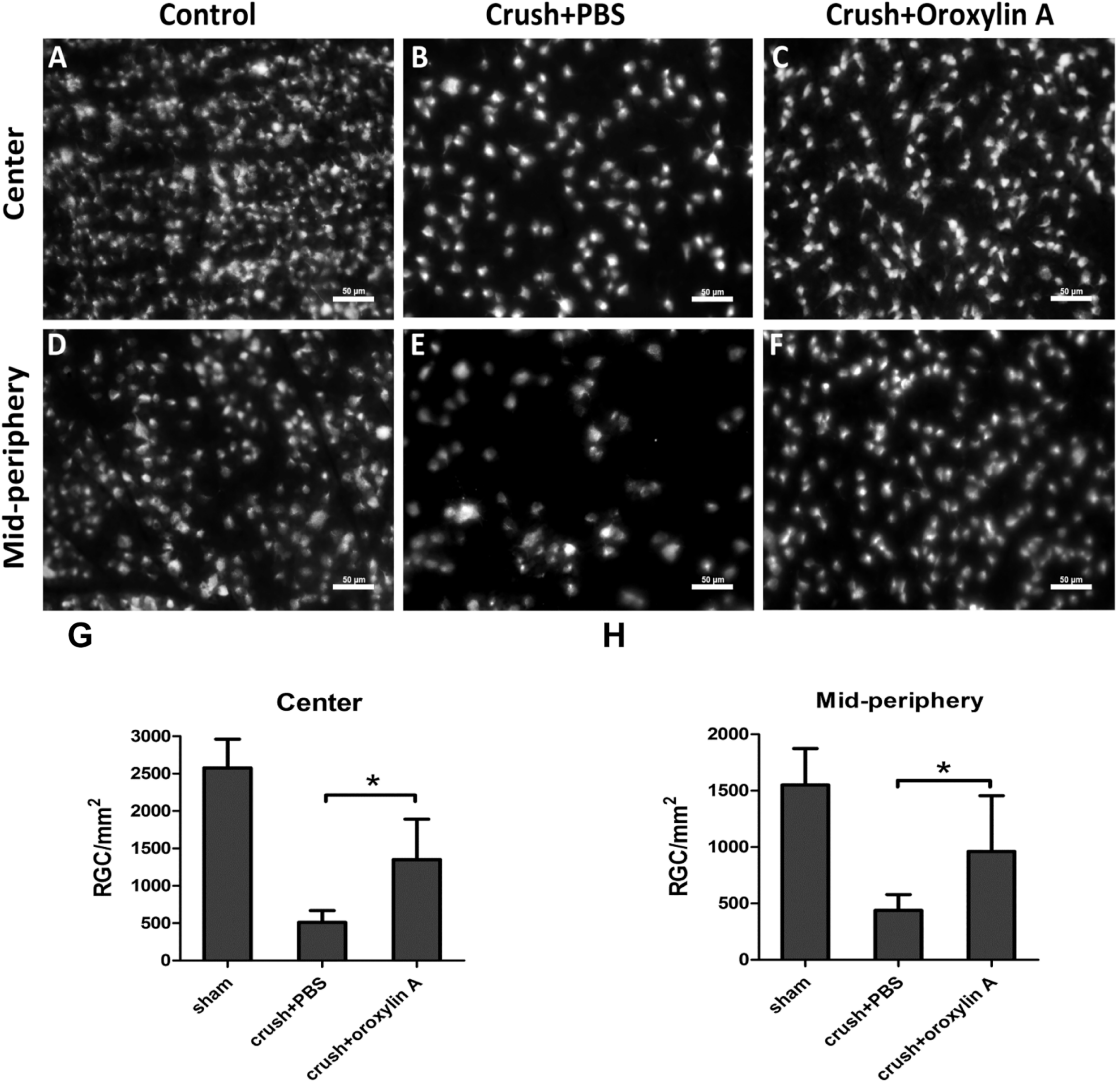
Improvement in RGC density in the retinas treated oroxylin A two weeks after ON crush. **(A, F)** RGC densities in the sham group were 2577 ± 383/mm^2^ and 1550 ± 325/mm^2^, respectively. Two weeks after ON crush, the densities of RGCs in the central retina of the **(B)** PBS-treated group and **(C)** oroxylin A-treated group were 510 ± 158/mm^2^ and 1352 ±476/mm^2^ respectively, and **(E, F)** in mid-peripheral retina were 439 ±139/mm^2^ and 960 ± 465/mm^2^ respectively, showing a significant preservative effect by oroxylin A (n = 6 in each group; p<0.05) *p<0.05.

In order to evaluate the effect of oroxylin A on RGC survival after crush injury to the optic nerve, we performed morphometric analysis of retrograde labeled RGCs of the rat retina in sham-operated group, crushed with PBS-treated group and crushed with oroxylin A-treated group. The densities of RGCs in the central and mid-peripheral retina in the sham-operated eyes were 2577 ± 383/mm^2^ and 1550 ± 325/mm^2^, respectively. Two weeks after ON crush, the densities of RGCs in the central retina of the oroxylin A-treated group and PBS-treated group were 1352 ±476/mm^2^ (52.5% survival) and 510 ± 158/mm^2^ (19.8% survival) respectively, and in mid-peripheral retina were 960 ± 465/mm^2^ (61.9% survival) and 439 ±139/mm^2^ (28.3% survival) respectively (**Fig 1**). There were significant differences in RGC densities in the oroxylin A-treated groups in both central and peripheral retinas compared with the PBS-treated group (n = 6 in each group, P<0.05). The results demonstrate that RGC survival rate increases by approximately 32.7% in the central retina and 33.6% in the mid-peripheral retina in the oroxylin A-treated group as compared to the PBS-treated group. These results clearly indicate that oroxylin A significantly promoted neuroprotection of RGC after ON crush.

**Fig 2:**
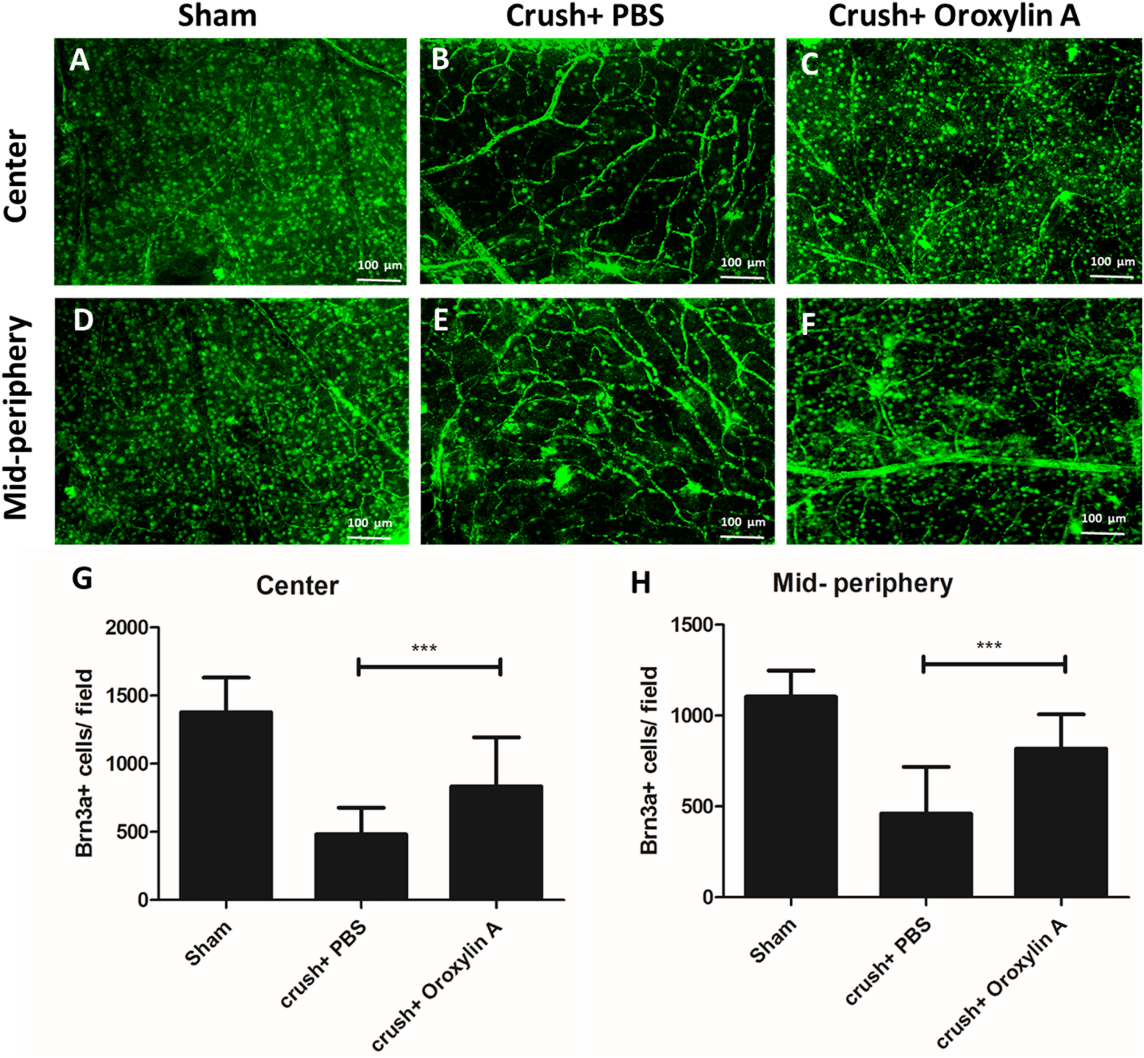
Retina flat mounts with Brn-3a validated RGC survival in Oroxylin-A treated group. Representative image of Brn-3a+ cells in the central retina (A-C) and mid-peripheral retina (D-F) in each group. The oroxylin-A treated group shows significantly higher Brn-3a+ cells compared to PBS treated group in the (G) central (894 ±367/mm^2^ vs 475± 203/mm^2^) and(H) mid-peripheral retina (800± 199/mm^2^ vs 474 ± 254/mm^2^) respectively. n = 6 in each group; ***p<0.001.

Further to validate the RGC survival rate we performed immunostaining in whole mount retina with Brn3a (**Fig 2**). The densities of Brn-3a+ cells in the central and mid-peripheral retina in the sham-operated eyes were 1378 ± 253/mm2 and 1095 ± 142/mm2 respectively. The densities of Brn-3a in the central retina of the oroxylin A-treated group and PBS-treated group were 847 ± 367/mm^2^ (64.9% survival) and 475 ±203/mm^2^ (34.5% survival) respectively, and in mid-peripheral retina were 801 ± 199/mm^2^ (73% survival) and 474 ±254/mm^2^ (43.2% survival) respectively (Fig 2). There were significant differences in RGC densities in the oroxylin A-treated groups in both central and peripheral retinas compared with the PBS-treated group (n = 6 in each group, P<0.001). The results demonstrate that RGC survival rate increases by approximately 30.4% in the central retina and 29.7% in the mid-peripheral retina in the oroxylin A-treated group as compared to the PBS-treated group. These results clearly indicate that oroxylin A significantly promoted neuroprotection of RGC after ON crush.

### Improvement in P1 amplitude in the oroxylin A–treated group

**Fig 3:**
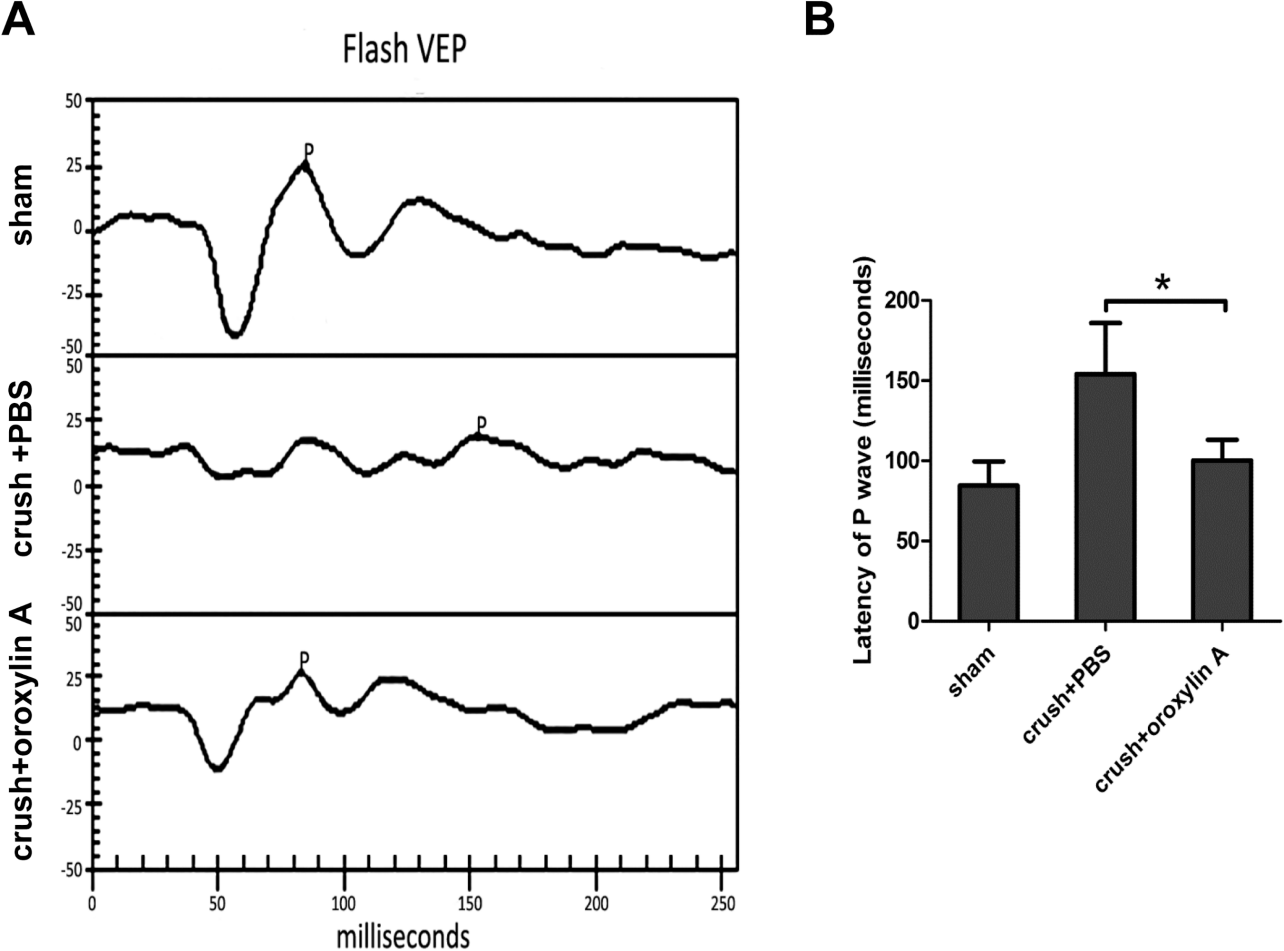
Improvement in the latency of the P1 wave in FVEPs after oroxylin A treatment. A. Representative flash VEP tracings at 2 weeks after ON crush. B. The latency of the P1 wave was 85 ± 15 ms, 154 ± 31 ms and 100 ± 12 ms in the sham, PBS-treated and oroxylin A-treated rats, respective Oroxylin A-treated group had shorter P1 latency than the PBS-treated group. (n = 6 in each group, p<0.05). *p<0.05.

To assess the visual function, we evaluated the changes of P1 latency in FVEP two weeks after ON crush. In the sham group, the latency of P1 wave was 85 ± 15 ms. In the PBS-treated group, the latency of the P1 delayed to 154 ± 31 ms. The latency of the P1 wave was 100 ± 12 ms in the oroxylin A-treated group (**Fig 3**). The latency of P1 wave was more significantly delayed in the PBS-treated group than in the oroxylin A-treated group (n = 6 in each group, p<0.05) The FVEP results demonstrate that the oroxylin A-treated group had significantly preserved visual function as compared to the PBS-treated group at 2 weeks after ON crush.

### Decreased number of TUNEL positive cells in the RGC layer of oroxylin A-treated retinas

**Fig 4:**
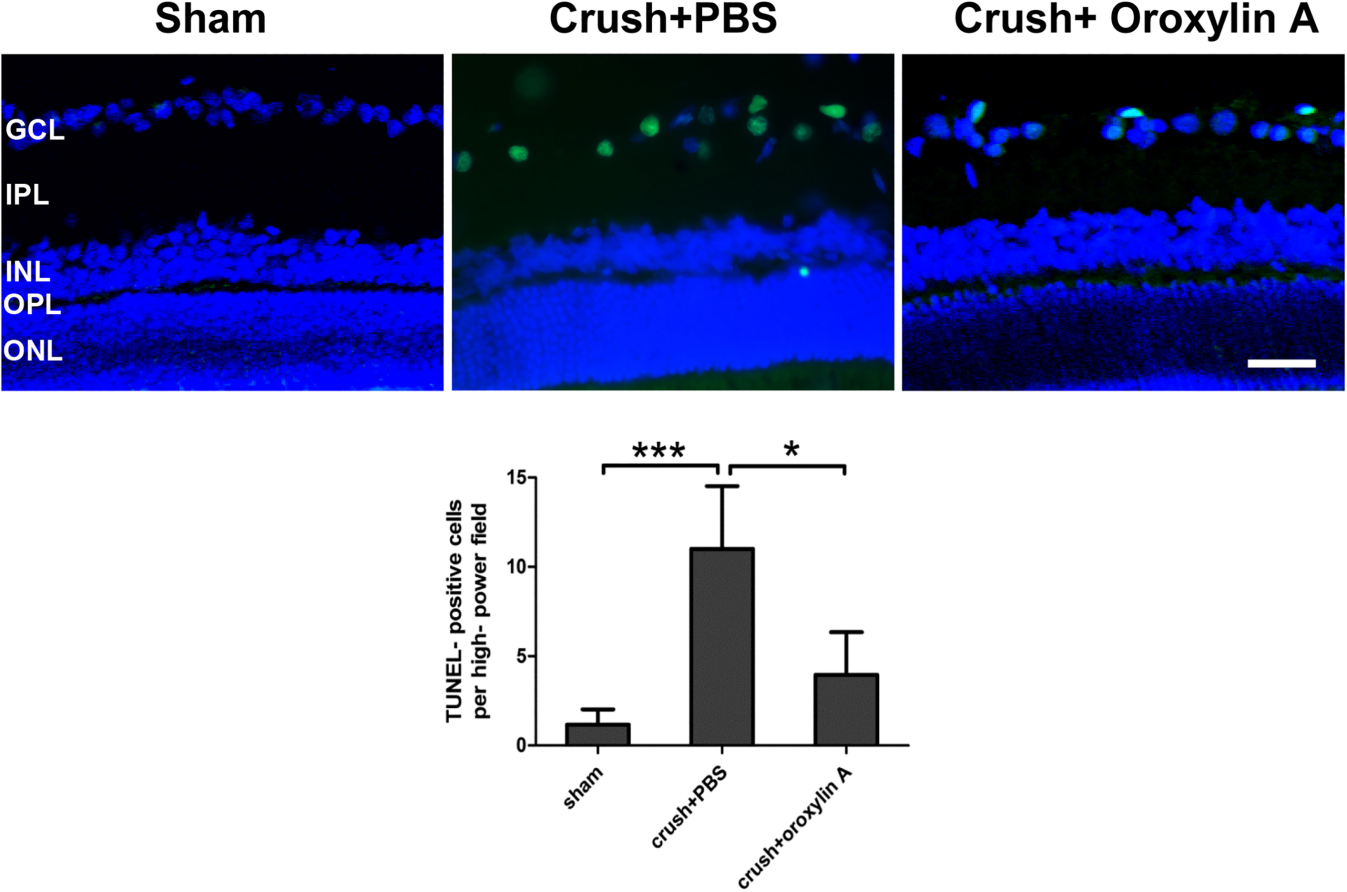
Assays of TUNEL revealed a decreased number of apoptotic cell after oroxylin A treatment. The upper column was reprehensive of the TUNEL in the retinas among the three groups. The lower column illustrates that there were 1.2 ± 0.9 positive cells/HPF in the RGC layers of retina in the sham-operated rats, 11.0 ± 3.5 positive cells/HPF in the PBS-treated group and 4.0 ± 2.4 positive cells/HPG in the oroxylin A-treated rats (n = 6 in each group). *p<0.05, ***p<0.001. Scale bar: 50μM. GCL, ganglion cell layer; INL, inner nuclear layer; IPL, inner plexiform layer; ONL, outer nuclear layer; OPL, outer plexiform layer.

TUNEL assay demonstrated that TUNEL positive cells/HPF (high powered field) was 1.2 ± 0.9 cells in the sham-operated rats, 11.0 ± 3.5 positive cells/HPF in the PBS-treated group (p<0.001 vs sham group) and 4.0 ± 2.4 positive cells/HPG in the oroxylin A-treated rats (p<0.05 vs. PBS-treated group) in the RGC layer (**Fig 4**). As shown in Fig 3, TUNEL-positive cell numbers were markedly increased in the RGC layer in the PBS-treated group and oroxylin A reduced the number of TUNEL positive cells, demonstrating that administration of oroxylin A had a significant anti-apoptotic effect on RGCs after ON crush.

### Oroxylin A reduced ED-1 positive cells in the ONs after ON crush

**Fig 5:**
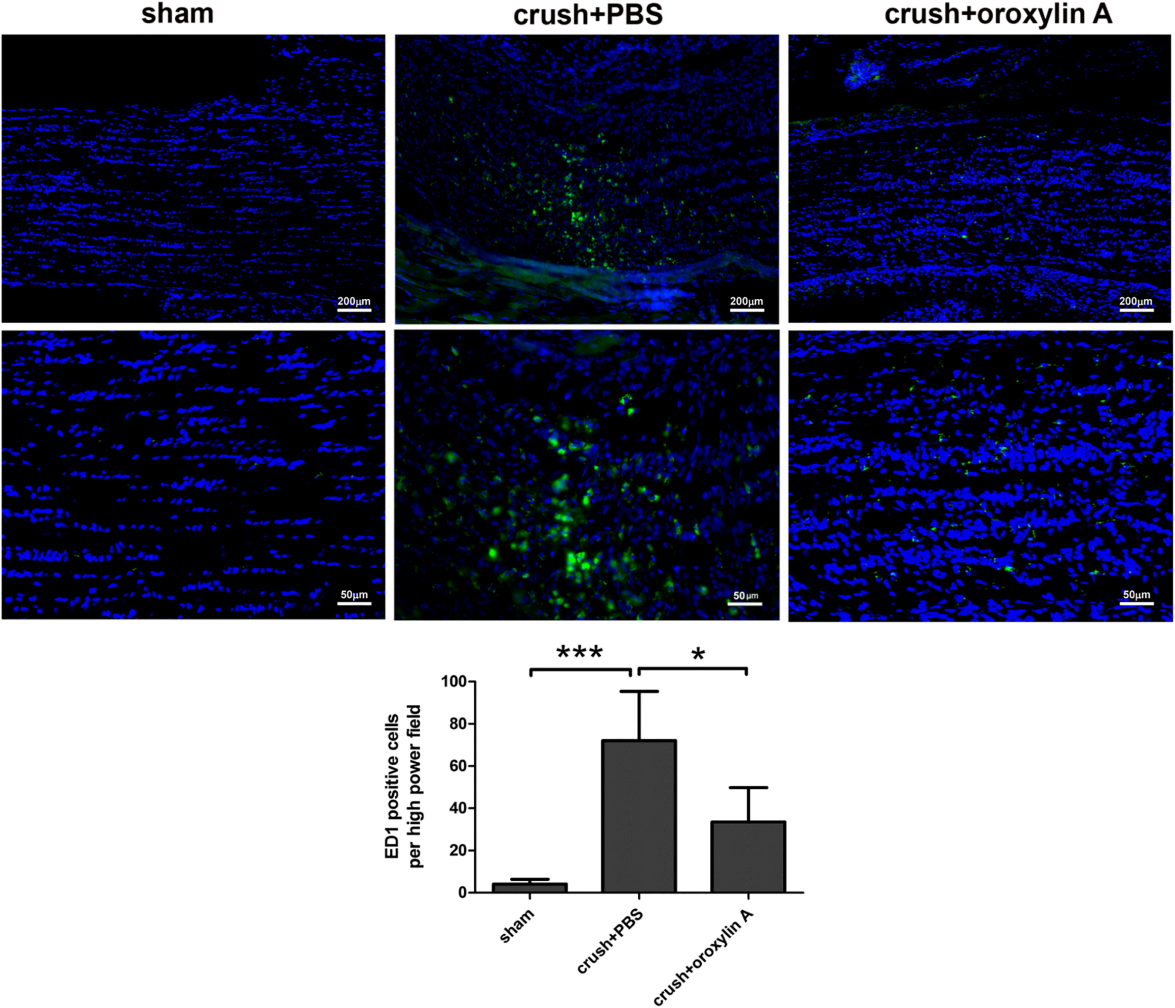
Less infiltration of ED1 in ONs treated with oroxylin A 2 weeks after ON crush. The upper column was representative of ED1 staining in the longitudinal sections of ON. The lower column indicates that the ED1 positive cells/HPF in the sham group, PBS-treated group and oroxylin A-treated group were 4.1 ± 2.2, 72.0 ± 23.3 and 33.5 ± 16.3, respectively. n = 6 in each group. *p<0.05, ***p<0.001.

At two weeks after ON crush insult, ED1 positive cells were prominent at the ON lesion sites in the PBS-treated group (72.0 ± 23.3 cells/HPF) and significantly less infiltration of ED1 positive cells/HPF in the ON after oroxylin A treatment (33.5 ± 16.3 cells/HPF) (**Fig 5**). These results indicate that oroxylin A administration had anti-inflammatory effects at the ON after insult, as demonstrated by less ED-1 labeled macrophage/microglial accumulation at ONs.

### Oroxylin A reduced the upregulation of GFAP in the retinal after crush injury

**Fig 6:**
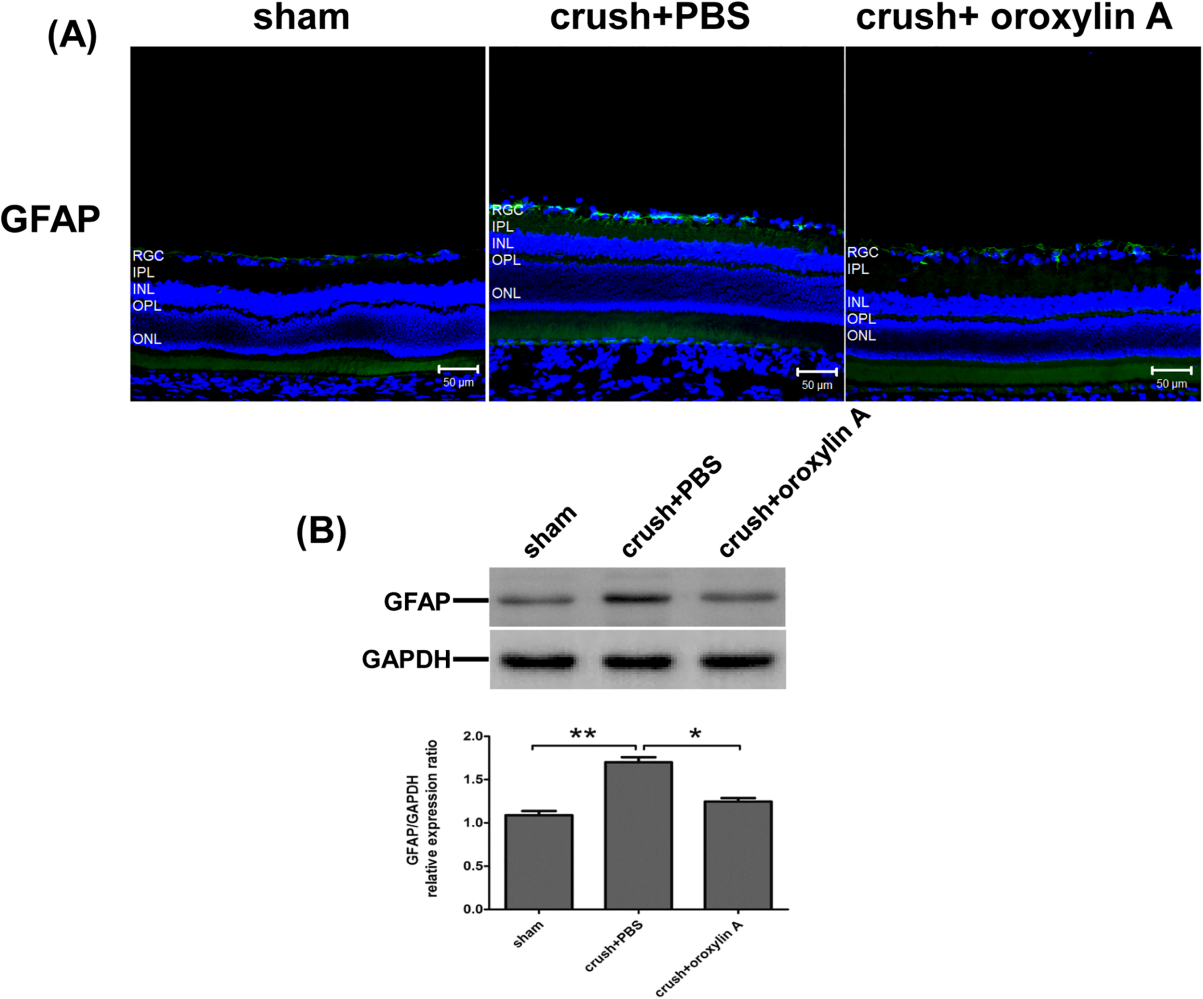
Oroxylin A attenuated retinal gliosis at 2 weeks after ON crush. (A) GFAP (astrocytes and Muller cells) immunoreactivity in retinal sections. Effects of oroxylin A on the suppression of GFAP level in the retina at 2 weeks after ON crush. (B) Western blotting showing the expression levels of GFAP in the retina. In the bar graph, the expression level of GFAP is expressed as a ratio to GAPDH expression Values for sham-operated retinas were set to 1. Results represent the means ± S.D for three independent experiments. *p<0.05. **p<0.01. Scale bar: 50μm.

GFAP is a sensitive marker for retinal gliosis in response to retinal neuronal degeneration [35, 36]. GFAP immunoreactivity is normally observed only in the astrocytes in the ganglion cell layer of normal retinas (**Fig 6**). Optic nerve crush injury resulted in significantly increased GFAP immunoreactive staining in the inner retina. Enhanced GFAP level was significantly reduced in the ON-crushed, oroxylin A-treated group. (p<0.05 when compared to ON-crushed, PBS-treated group).

### Suppression of pro-inflammatory cytokines, iNOS, and COX-2 expression

**Fig 7:**
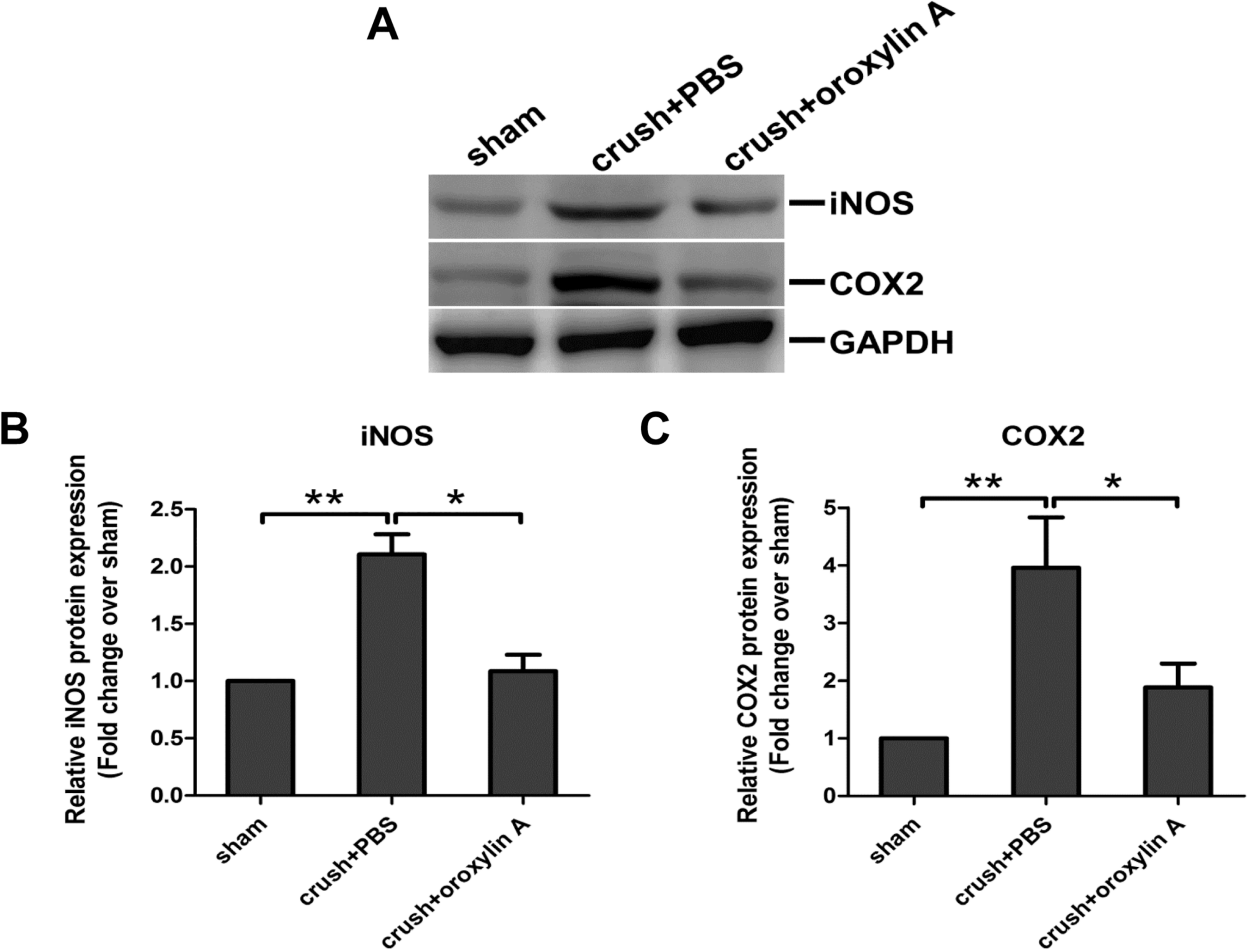
Reducing the expression level of iNOS and COX-2 in the retinas treated with oroxylin A after ON crush. (A) Effects of oroxylin A on suppression of iNOS and COX-2 in the retina at 2 weeks after ON crush. (B.C) Quantitative analysis of (A). In the bar graph, the expression level of iNOS and COX-2 are expressed as a ratio to GAPDH expression Values for sham-operated retinas were set to 1. Results represent the means ± S.D for three independent experiments. *p<0.05.

The expression level of iNOS and COX-2 (**Fig 7**) was significantly elevated in the PBS-treated retinas at two weeks after ON crush. Oroxylin A treatment suppressed the increase in iNOS and COX-2 expression induced by optic nerve injury. These results indicate that oroxylin A attenuates the elevated pro-inflammatory cytokines, iNOS and COX-2 expression in the retina after ON crush injury.

## Discussion

This study demonstrated that oroxylin A has a neuroprotective effect in a rat model subject to ON crush. The survival rate of RGCs was detected using retrograde Fluorogold labeling provided morphologic evidence that oroxylin A promoted RGCs survival after ON crush and it was further validated by Brn-3a immunostaining though RGC density differed in both experiments which might be a result of different staining efficiency. In addition, oroxylin A also exerted beneficial effect on preserving the visual function as demonstrated by FVEP after crush injury.

Previous studies showed an important role of iNOS and COX-2 in the pathogenesis of RGC loss after crush injury [15, 17, 19, 37]. NO produced by iNOS in activated inflammatory cells, microglia or injured neurons contributes to cytotoxicity resulting in neuron death and axonal damage [38, 39]. COX-2 is a key player in neuroinflammation signaling, which has also been implicated to involve in the apoptotic death of neurons [40, 41]. Our TUNEL assay results showed that the administration of oroxylin A was anti-apoptotic on RGCs after ON crush injury. We also demonstrated that oroxylin A significantly reduced expression of iNOS and COX-2 in the ON crushed-retina. These observations may suggest that suppression of the crush insult-induced iNOS and COX-2 expression may contribute to the anti-apoptotic effect of oroxylin A. The neuroprotective effect of oroxylin A showed in this study might be accomplished by decreasing expression of the pro-inflammatory factors and further reducing apoptosis of RGCs.

Earlier, we demonstrated that ED-1 positive cells accumulated at the crush site [28, 30] and increasing evidence suggests that nerve injury can initiate iNOS and COX-2 expression in macrophage/microglia at the injury site [17, 21, 28, 42]. Furthermore, prolonged and massive activation of microglia and astroglia with excessive amounts of pro-inflammatory mediators release has been observed during the pathogenesis of neuronal death in CNS injury [43, 44]. Immediate administration of oroxylin A decreased the accumulation of ED-1 positive macrophage/microglia at the ON lesion site, reduced retinal gliosis and suppressed the expression of iNOS and COX-2 in the retina after crush injury. Furthermore, administration of oroxylin A was demonstrated neuroprotective effects by reducing microglia activation and promoting neuron survival in several studies [24–27]. Therefore, the neuroprotective effect of oroxylin A showed in this study might be accomplished by inhibiting microglial activation and thereby decreasing the release of neurotoxic cytokines and further reducing apoptosis of RGCs.

In conclusion, systemic administration of oroxylin A promotes RGCs survival and improves visual function after optic nerve crush. The oroxylin A mechanisms of action may include anti-apoptotic effect by decreasing TUNEL-positive cells and modulation of neuroinflammation by blocking microglia activation, reactive gliosis and the induction of iNOS and COX2 in the damaged tissue. Our results support the therapeutic potential of oroxylin A for ischemic or traumatic optic neuropathies.

## Supporting information

S1 Table Summary of the rats used and the study design. (TIF)
