# Supplementary figures and images for: Oroxylin A promotes retinal ganglion cell survival in a rat optic nerve crush model
Source: PLoS One. 2017 Jun 22;12(6):e0178584. doi: 10.1371/journal.pone.0178584 (PMC5480866; doi:10.1371/journal.pone.0178584)

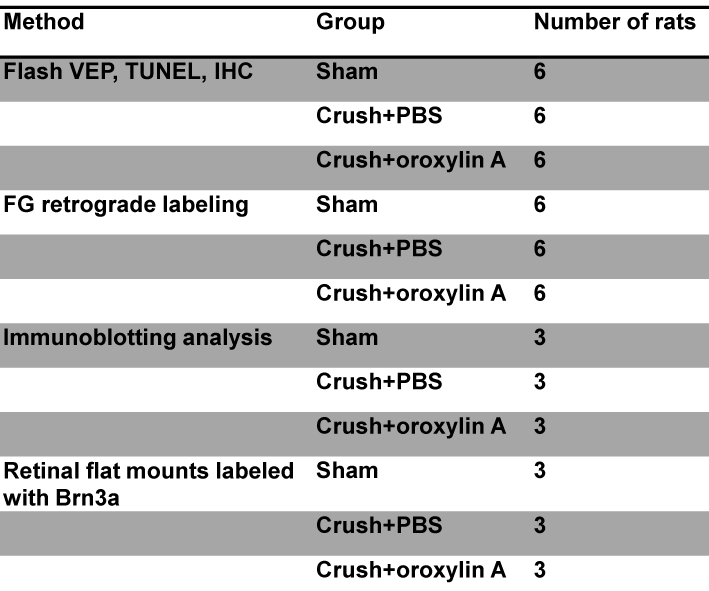

Supplement: S1 Table — (TIF) [file pone.0178584.s001.tif]
